# RNA-Seq Reveals Infection-Induced Gene Expression Changes in the Snail Intermediate Host of the Carcinogenic Liver Fluke, *Opisthorchis viverrini*

**DOI:** 10.1371/journal.pntd.0002765

**Published:** 2014-03-27

**Authors:** Sattrachai Prasopdee, Javier Sotillo, Smarn Tesana, Thewarach Laha, Jutharat Kulsantiwong, Matthew J. Nolan, Alex Loukas, Cinzia Cantacessi

**Affiliations:** 1 Food-borne Parasite Research Group, Department of Parasitology, Faculty of Medicine, Khon Kaen University, Khon Kaen, Thailand; 2 Centre for Biodiscovery and Molecular Development of Therapeutics, Australian Institute of Tropical Health and Medicine, James Cook University, Cairns, Queensland, Australia; 3 Department of Biology, Faculty of Science, Udon Thani Rajabhat University, Udon Thani, Thailand; 4 Department of Veterinary Medicine, University of Cambridge, Cambridge, United Kingdom; Johns Hopkins Bloomberg School of Public Health, United States of America

## Abstract

**Background:**

*Bithynia siamensis goniomphalos* is the snail intermediate host of the liver fluke, *Opisthorchis viverrini*, the leading cause of cholangiocarcinoma (CCA) in the Greater Mekong sub-region of Thailand. Despite the severe public health impact of *Opisthorchis*-induced CCA, knowledge of the molecular interactions occurring between the parasite and its snail intermediate host is scant. The examination of differences in gene expression profiling between uninfected and *O. viverrini*-infected *B. siamensis goniomphalos* could provide clues on fundamental pathways involved in the regulation of snail-parasite interplay.

**Methodology/Principal Findings:**

Using high-throughput (Illumina) sequencing and extensive bioinformatic analyses, we characterized the transcriptomes of uninfected and *O. viverrini*-infected *B. siamensis goniomphalos*. Comparative analyses of gene expression profiling allowed the identification of 7,655 differentially expressed genes (DEGs), associated to 43 distinct biological pathways, including pathways associated with immune defense mechanisms against parasites. Amongst the DEGs with immune functions, transcripts encoding distinct proteases displayed the highest down-regulation in *Bithynia* specimens infected by *O. viverrini*; conversely, transcription of genes encoding heat-shock proteins and actins was significantly up-regulated in parasite-infected snails when compared to the uninfected counterparts.

**Conclusions/Significance:**

The present study lays the foundation for functional studies of genes and gene products potentially involved in immune-molecular mechanisms implicated in the ability of the parasite to successfully colonize its snail intermediate host. The annotated dataset provided herein represents a ready-to-use molecular resource for the discovery of molecular pathways underlying susceptibility and resistance mechanisms of *B. siamensis goniomphalos* to *O. viverrini* and for comparative analyses with pulmonate snail intermediate hosts of other platyhelminths including schistosomes.

## Author Summary

Despite recent significant advances in knowledge of the fundamental biology of the carcinogenic liver fluke *Opisthorchis viverrini*, little is known of the complement of molecular interactions occurring between this parasite and its prosobranch snail intermediate host, *Bithynia siamensis goniomphalos*. The determination of such interactions is a key, necessary component of the development of future integrated control strategies for liver fluke infection and associated bile duct cancer. Here, we use cutting-edge high-throughput sequencing technologies and advanced bioinformatic analyses to characterize, for the first time, qualitative and quantitative differences in gene expression between uninfected and *O. viverrini*-infected *B. siamensis goniomphalos* collected from an endemic region of Northeast Thailand. The analyses led to the identification of a number of molecules putatively involved in immune defense pathways against invading *O. viverrini*, and of key biological mechanisms potentially implicated in the ability of the parasite to successfully colonize its snail intermediate host. We believe that this ready-to-use molecular resource will provide the scientific community with new tools for the development of strategies to control the spread of liver fluke infection and the resulting bile duct cancer.

## Introduction

Cholangiocarcinoma (CCA, or bile-duct cancer) is one of the most common malignant cancers in Southeast Asia, with incidence rates ranging from 93.8 to 317.6 per 100,000 people/year in some districts of Northeast Thailand alone [1], [2]. Chronic infections by the food-borne human liver fluke *Opisthorchis viverrini* have been associated with the occurrence of CCA in several Southeast Asian countries (including Thailand and Laos) [2], [3], which has led to the inclusion of this parasite amongst the Group 1 carcinogens by the International Agency for Research on Cancer [4]. Typically, CCA caused by *O. viverrini* is diagnosed 30–40 years after primary infection, and death occurs within 3–6 months post-diagnosis [5]. The life cycle of *O. viverrini* is complex, involving two intermediate hosts and multiple developmental stages [6]. Briefly, piscivorous definitive hosts, including humans, shed the embryonated eggs through the faeces; in aquatic environments, eggs are ingested by freshwater prosobranch snails of the genus *Bithynia*, in which the motile embryos ( = miracidia) hatch and develop into sporocysts. In the body of the first intermediate hosts, the sporocysts undergo asexual reproduction through the stages of rediae and cercariae. Subsequently, the motile cercariae are released into the aquatic environment where they actively seek the second intermediate hosts, i.e. cyprinid fishes (e.g. *Puntius* spp.). Upon penetrating the skin of these fishes, the parasites encyst as metacercariae under the skin and/or in muscular tissue. Humans acquire the infection by ingesting raw or undercooked infected fishes [5], [6]. Following ingestion, the metacercariae excyst in the duodenum and migrate as juvenile flukes to the intra-hepatic bile ducts, where they develop to adult hermaphrodite flukes within ∼4 weeks. The sexually mature adults release eggs that, *via* the bile, are excreted with the faeces into the environment [6].

Over the last decade, much effort has been directed towards the elucidation of the molecular mechanisms that determine the occurrence of CCA in *O. viverrini*-infected individuals [2], [7]–[9], and advances in high-throughput sequencing technologies and bioinformatics are contributing towards a better understanding of the systems biology of this parasite [10], [11]. While these advancements undoubtedly provide a foundation for the development of novel strategies for the diagnosis, treatment and prevention of *O. viverrini*-induced CCA [3], long-term control of this devastating disease strictly relies on the development of integrated approaches, targeting the parasite as well as its intermediate hosts [12], [13]. Thus far, large-scale molecular investigations of snail species acting as intermediate hosts of human parasites have mainly involved pulmonate species of the genus *Biomphalaria*, which harbor *Schistosoma* blood flukes (see *Biomphalaria glabrata* Genome Initiative at http://biology.unm.edu/biomphalaria-genome/index.html; [14], [15]) while, until recently, knowledge of the fundamental molecular biology of prosobranch snails has remained limited [12], [13]. Our group has recently utilized high-throughput (Illumina) sequencing and advanced bioinformatic analyses of sequence data to generate and characterize the first reference transcriptome of *Bithynia siamensis goniomphalos* (Gastropoda, Bithyniidae), the snail intermediate host of *O. viverrini* in areas of Northeast Thailand, Lao PDR, Cambodia and South Vietnam [13]. This sequence dataset represents a valid resource for molecular studies of these organisms and provides a solid basis for investigations of snail-parasite interactions at the molecular level. In the present study, we employ high-throughput (Illumina) sequencing and bioinformatic technologies to characterize differences in levels of gene transcription between uninfected and *O. viverrini*-infected *B. siamensis goniomphalos* and identify key molecules and biological pathways inferred to be associated with the processes of invasion and survival of the parasite in the snail intermediate host.

## Materials and Methods

### Procurement of snail materials and assessment of infection status

Adult *B. siamensis goniomphalos* snails were collected from natural freshwater bodies in Amphoe Muang, Khon Kaen Province, Thailand. The soft tissues of each snail were removed from the shells and washed three times with sterile distilled water. A portion of the hepatopancreas was isolated from each snail and subjected to DNA extraction using the Direct PCR kit (Thermo Scientific) according to manufacturer's instructions, while the remaining material was preserved in RNAlater (Sigma) for subsequent extraction of total RNA. A PCR targeting the *O. viverrini* pOV-A6 gene was employed to screen individual snails for infection by the liver fluke following the amplification protocol described by Sermswan et al. [16]. Amplicons were visualized on a 1.5% agarose gel stained in ethidium bromide and the presence of bands of approximately 330 bp in size was considered diagnostic for *O. viverrini* infection [17].

### RNA isolation and Illumina sequencing

Two distinct pools of soft tissues from uninfected (UB; n = 4) and *O. viverrini*-infected snails (IB; n = 4) were subjected to isolation of total RNA using the Trizol reagent (Invitrogen), followed by DNAse I treatment (Promega) according to the manufacturer's instructions. The amounts and integrity of total RNA were determined using a 2100 BioAnalyzer (Agilent Technologies). Polyadenylated (PolyA+) RNA was purified from 10 µg of total RNA from each pool of uninfected and *O. viverrini*-infected snails using Sera-Mag oligo(dT) beads, fragmented to a length of 100–500 nucleotides and reverse transcribed to cDNA using random hexamers. The size-fractionated cDNA was end-repaired and adaptor-ligated according to the manufacturer's protocol (Illumina). Ligated products of ∼300 bp were excised from agarose gels and PCR-amplified (15 cycles) [18]. Products were cleaned using a MinElute column (Qiagen, Netherlands) and paired-end sequenced on an Illumina HiSeq 2000 [19] according to the manufacturer's protocol.

### Bioinformatic analyses

Following removal of adapter sequences and sequences with suboptimal read quality (i.e., PHRED score of 32.0), the remaining 90 bp single-read sequences generated from the non-normalized cDNA libraries from UB and IB, as well as the raw sequence data generated previously [13] were pooled and assembled using the program Trinity [20] (http://trinityrnaseq.sourceforge.net/). Briefly, a representative set of transcripts was assembled using the software Inchworm; next, the Chrysalis software was used to cluster portions of alternatively spliced transcripts and/or unique portions of paralogous genes and construct a de Brujin graph for each cluster of transcripts. Finally, each de Brujin graph was analysed and alternatively spliced isoforms and transcripts derived from paralogous genes were resolved using the Butterfly software [20] (http://trinityrnaseq.sourceforge.net/). The non-redundant, assembled dataset was subsequently compared to transcriptomic sequence data available for *O. viverrini* [10], [11] as well as the entire genome sequences of *Clonorchis sinensis*, *S. mansoni*, *S. japonicum* and *S. haematobium* [10], [21]–[24]; sequences with high similarity to known *O. viverrini* and/or other molecules from parasitic trematodes (e-value cut-off: 1e-15) were excluded [25]. The non-redundant transcriptomic dataset for *B. siamensis goniomphalos* was then annotated using an established approach [23], [25]. Briefly, assembled contigs were compared using the BLASTn and BLASTx algorithms [26] to sequences available in the nucleotide sequence collection (nt) of NCBI (www.ncbi.nlm.nih.gov), and in the non-redundant (nr) (www.ncbi.nlm.nih.gov), Swiss-Prot (http://expasy.org/), Kyoto Encyclopedia of Genes and Genomes (KEGG; http://www.genome.jp/kegg/) and Clusters of Orthologous Groups (COG; http://www.ncbi.nlm.nih.gov/COG/) databases, respectively (e-value<0.00001), to identify putative homologues in other mollusks and organisms other than mollusks. In addition, the non-redundant *B. siamensis goniomphalos* transcriptome was compared, using a BLASTn algorithm, to an in-house built nucleotide sequence database containing 86,837 expressed sequence tags (ESTs) available for *B. glabrata* (http://www.ncbi.nlm.nih.gov/nucest/?term=biomphalaria+glabrata).

Proteins with the highest sequence similarity to conceptually translated *B. siamensis goniomphalos* transcripts were assigned Gene Ontology terms (GO; http://www.geneontology.org/) [27], according to the categories ‘Biological Process’, ‘Cellular Component’ and ‘Molecular Function’, using the Blast2GO software [28]. *B. siamensis goniomphalos* contigs with no homologues in available protein databases were conceptually translated using the ESTScan software and annotated using the same approach [29].

The short reads from each UB and IB dataset were then separately mapped to the non-redundant sequence data using SOAPAligner, such that each read was mapped to a unique transcript with a minimum alignment overlap of 40 nucleotides and a maximum of three mismatches per read. Reads that mapped to more than one transcript (“multi-reads”) were randomly allocated to a unique transcript, such that they were mapped only once. Comparative analyses of gene transcription levels between UB and IB were performed using the fragments per kilobase of exon per million fragments method (i.e., FPKM) [30]. The analysis of statistical difference of transcription was determined using a method developed for serial analysis of gene expression (SAGE) and applied to RNA-seq data [31]. Statistical significance was set at a *p* value of ≤0.01 and, to control for errors associated with multiple pairwise comparisons, a false-discovery rate (FDR) correction was applied to the data set [32]. GO and KEGG functional enrichment analyses for *B. siamensis goniomphalos* genes whose transcription levels were altered by infection with the parasite, were performed using the software GO::TermFinder with multiple testing correction using the Benjamini & Hochberg calculations of FDR (cut-off: FDR<0.01) [33], [34].

## Results

A total number of 60,210,044 and 56,426,082 raw reads were generated from UB and IB, respectively; of these, 55,137,440 (92%) and 52,476,468 (92%), respectively, were retained for further analyses (Table 1). All single reads generated in the present study have been deposited in the Sequence Read Archive (SRA) database of NCBI (http://www.ncbi.nlm.nih.gov/sra) under accession numbers SRR1046838 (UB) and SRR1046839 (IB), respectively. The assembly yielded 43,259 contigs, of which 20,171 displayed significant sequence similarity (e-value cut-off: 1e-15) to publicly available sequences from *O. viverrini*, *C. sinensis*, *S. mansoni*, *S. japonicum* and/or *S. haematobium* (data not shown) and were therefore excluded from subsequent analyses. All remaining 23,088 contigs (average length = 775 nt±679; maximum length = 12,119 nt; minimum length = 200; N50 = 671; cf. Table 1), matched previously assembled sequence data for *B. siamensis goniomphalos*, and displayed significant sequence similarity (e-value cut-off: 1e-05) with available ESTs from *B. glabrata* (not shown). Of the assembled sequence dataset, 5,461 (23.6%) and 13,283 (57.5%) contigs had homologues in the non-redundant nucleotide (nt) and protein (nr) sequence databases of NCBI, respectively (e-value cut-off: 1e-05; Table 1). Of the conceptually translated contigs with significant similarity to sequences in the nr database, 18.4% matched known proteins from the bivalve mollusk *Crassostrea gigas* (Table S1). This Transcriptome Shotgun Assembly Project has been deposited at DDJB/EMBL/GenBank under the accession GAQQ00000000.

**Table 1:** Summary of the RNA-Seq data from uninfected and *Opisthorchis viverrini*-infected *Bithynia siamensis goniomphalos* snails (UB and IB, respectively), prior and following assembly, and results of bioinformatic analyses and annotation.

*Raw reads (paired-end)*	60,210,044 (UB); 56,426,082 (IB)
*Assembly*	
Contigs (average length ± SD; min–max length)	23,088(775±679)
N50	671
GC content (%)	46.32
*Annotation*	
Contigs containing an Open Reading frame (%)	17,849 (77.3)
*Inferred via BLAST alignment*	16,398
*ESTScan predictions*	1,451
Returning SwissProt hits (%)	10,761 (60.3)
Gene Ontology (%)	5,950 (33.3)
*Number of parental Biological Process terms*	23
*Cellular component*	16
*Molecular function*	17
Returning a KEGG result (%)	9,497 (53.2)
*Number of predicted biological pathways*	259
Returning a COG result (%)	5,287 (29.6)
*Number of predicted functional categories*	25
*DEGs*	
Total number of DEGs (%)	7,655 (33)
Up-regulated in UB	735
Up-regulated in IB	6,920

The number of statistically significant (*p*≤0.01) differentially expressed genes (DEGs) in each UB and IB is also reported.

Of the 17,849 contigs that encoded a predicted protein (cf. Table 1), 10,761 (60.3%) had homologues in the Swiss-Prot database (cf. Table 1); these included mucins (n = 378), histones (n = 318), collagens (n = 251) and serine/threonine protein phosphatases (STPs; n = 181) and kinases (STKs; n = 119) (Table S1). A total of 5,950 predicted proteins (33.3%) could be assigned GO terms, while 9,497 (53.2%) matched homologous proteins in the KEGG database associated to 259 distinct biological pathways (Table 1). The 5,287 (29.6%) predicted proteins with matches in the COG database could be assigned to at least one of 25 functional categories, of which ‘general function prediction’ (84%), ‘translation, ribosomal structure and biogenesis’ (50.2%) and ‘post-translational modification, protein turnover and chaperones’ (41%) were the most represented (Tables 1 and S1).

**Figure 1:**
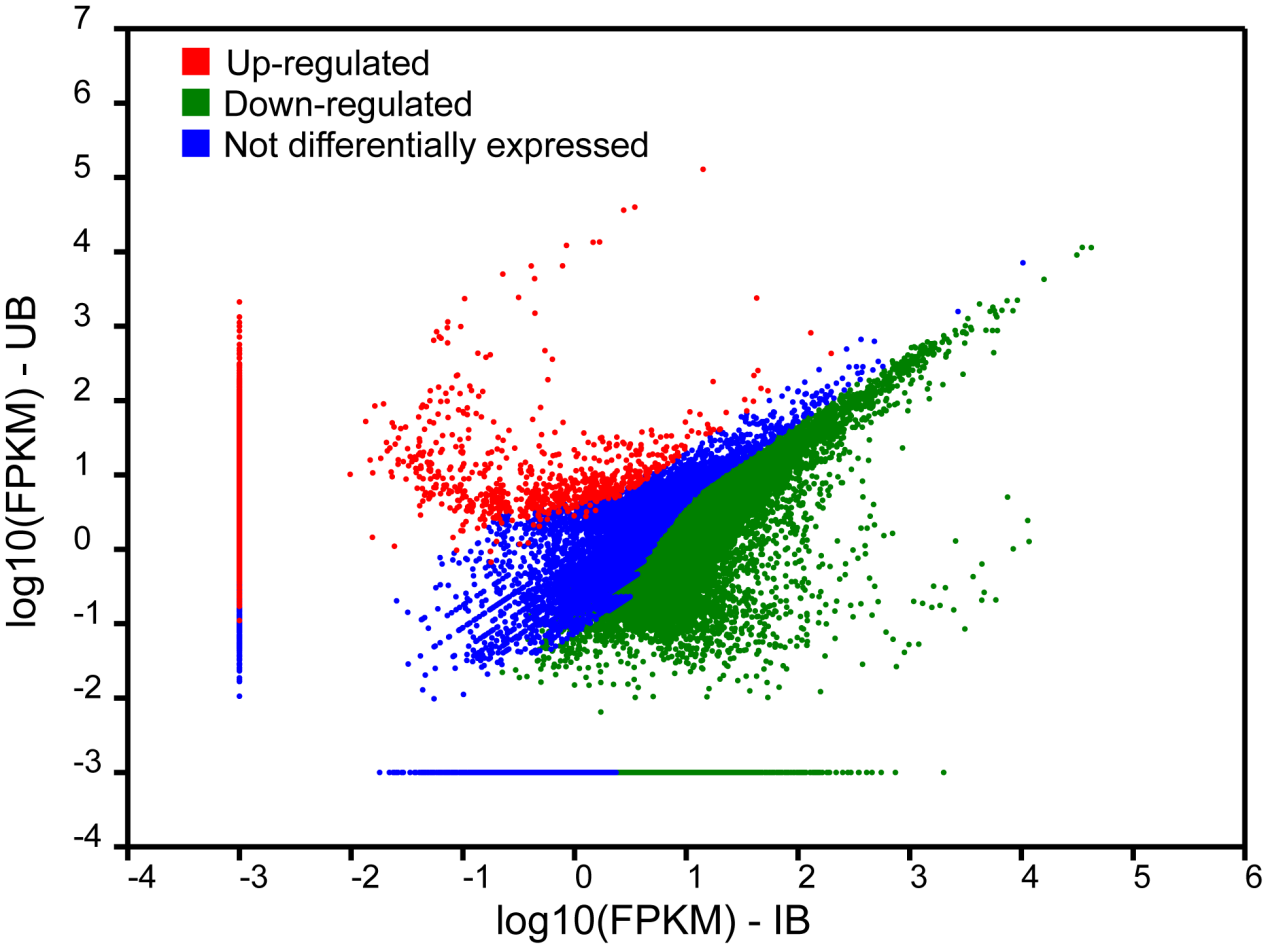
Scatter plot comparing log2 ratios of FPKM expression values for uninfected and *Opisthorchis viverrini*-infected *Bithynia siamensis goniomphalos* snails (UB and IB, respectively).

**Figure 2:**
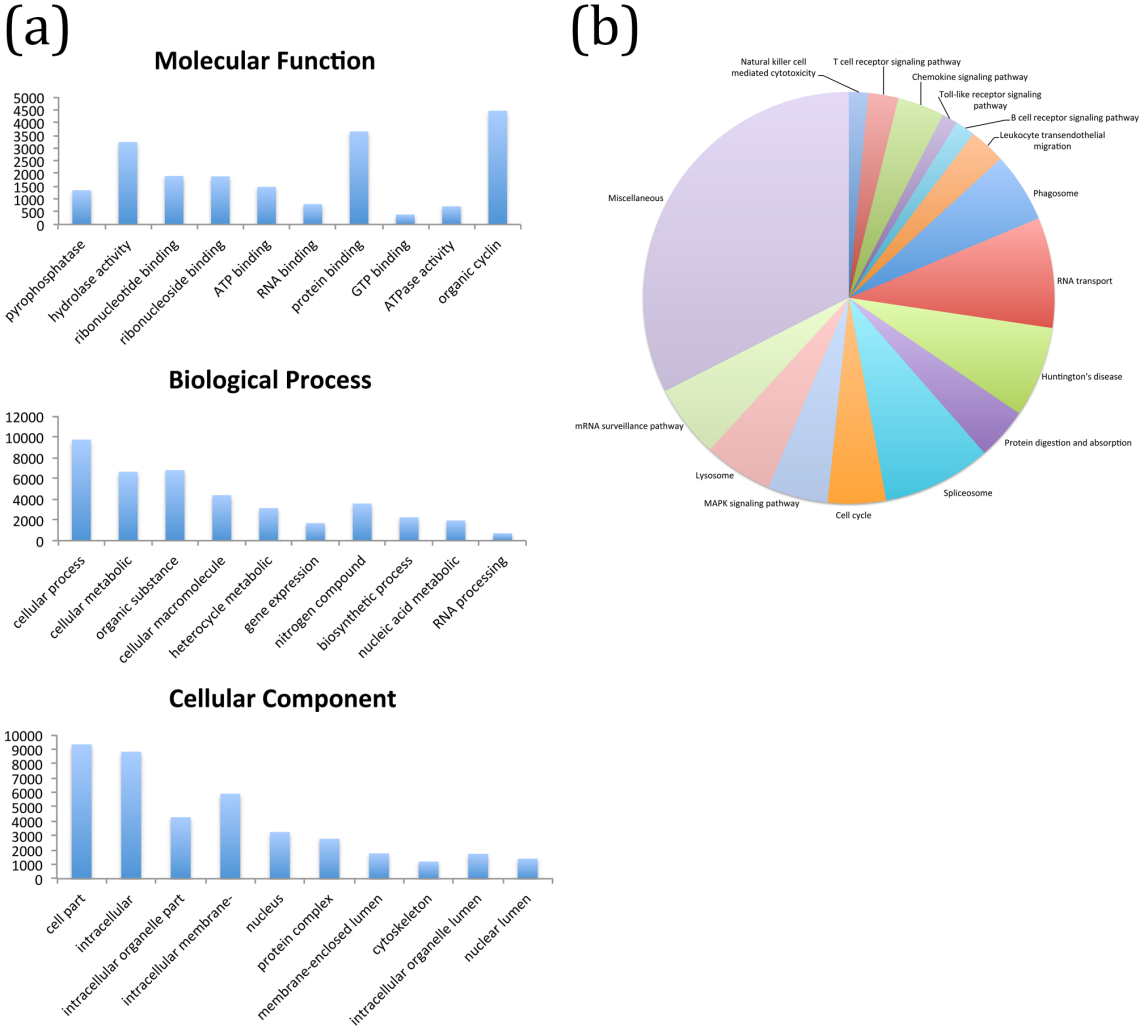
(a) Enriched Gene Ontology (GO) terms assigned to differentially expressed genes (DEGs) in uninfected and *Opisthorchis viverrini*-infected *Bithynia siamensis goniomphalos* snails (UB and IB, respectively), according to the categories ‘biological process’, ‘cellular component’ and molecular function; (b) enriched KEGG biological pathways assigned to DEGs in UB and IB, respectively.

**Figure 3:**
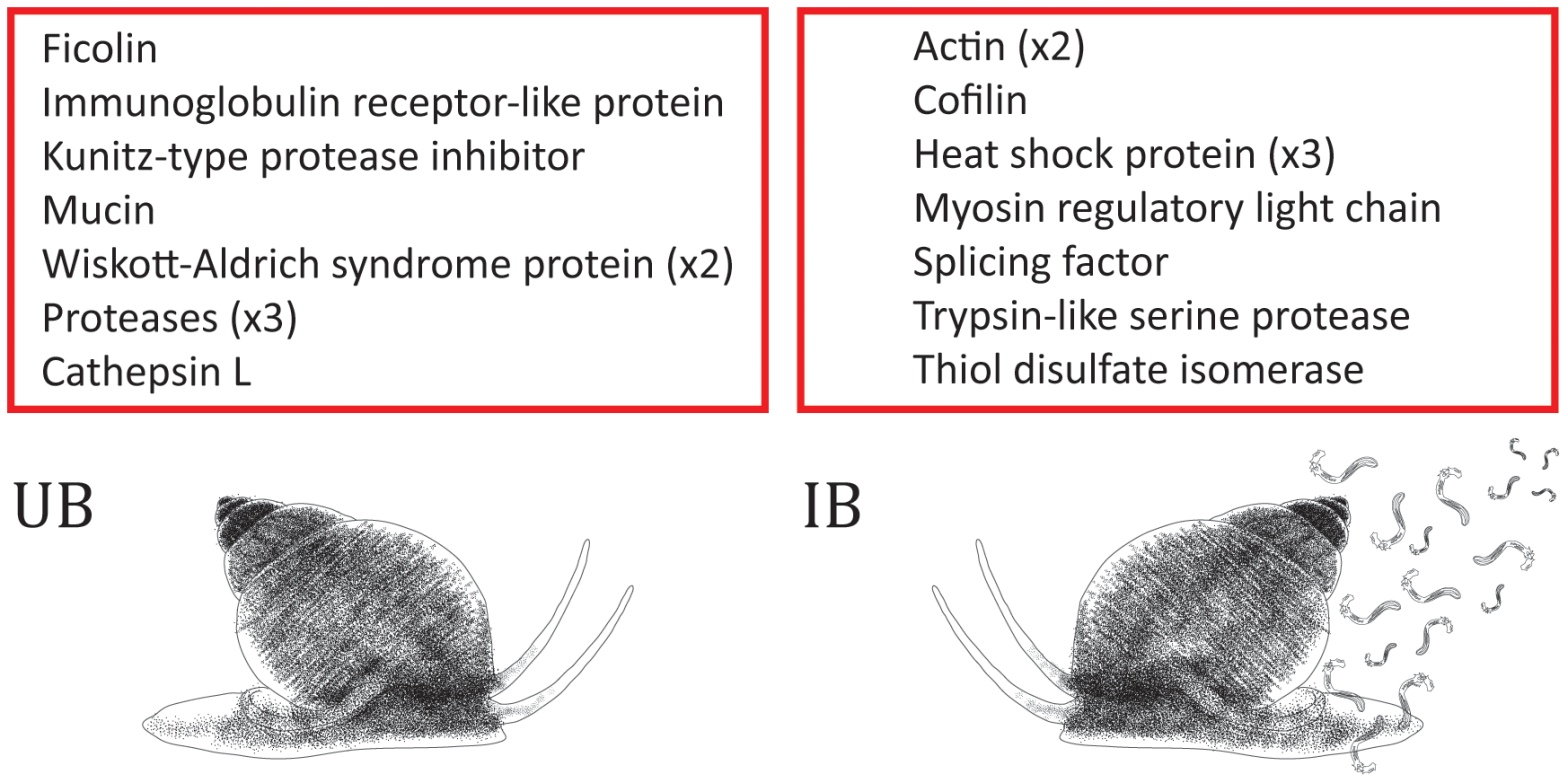
The top ten up-regulated genes linked to biological pathways associated to immune responses [based on orthology to molecules in the Kyoto Encyclopedia of Genes and Genomes (KEGG)] in each uninfected and *Opisthorchis viverrini*-infected *Bithynia siamensis goniomphalos* snails (UB and IB, respectively).

Comparative analyses of levels of gene expression revealed 6,920 and 735 DEGs in UB and IB, respectively (FPKM≥2.0; Table 1, Figure 1). Of the entire complement of molecules up-regulated in UB, 4,488 (64%) did not match known proteins in the nr database (Table S2); of the remaining 2,462, 7% were mucins, followed by uncharacterized proteins (2%) and immunoglobulin receptor homologues (0.6%). Of the 208 DEGs in IB with known homologues in the nr database (cf. Table S2), 38% were uncharacterized proteins, followed by actins (1%) and transcription factors (0.7%) (Table S2). Two haemocyanins and a Kunitz-type protease inhibitor were amongst the most significantly up-regulated DEGs in UB, whereas a histone and dynein displayed the highest expression levels in IB (Table 2). GO-enrichment analysis of DEGs in UB and IB revealed significant enrichment of the GO terms ‘cellular process’ (80.8% of DEGs), ‘organic substance metabolic process’ (53.3%) and ‘cellular metabolic process’ (52.1%) within ‘biological process’, ‘cell’ (90.4%), ‘intracellular part’ (85.4%) and ‘organelle’ (68.7%) within ‘cellular component’ and ‘protein binding’ (28.3%), ‘hydrolase activity’ (25.1%) and ‘ribonucleoside binding’ within ‘molecular function’ (Figure 2a). KEGG pathway enrichment analysis identified a total of 43 pathways linked to DEGs, including ‘RNA transport’ (8.6%), ‘Huntington’s disease’ (7.1%) and ‘spliceosome’ (8.5%) (Figure 2b). Out of 375 DEGs predicted to be involved in biological pathways linked to immune responses (cf. Figure 2b), homologues of metalloproteases, and actins and heat shock proteins displayed the largest up-regulation in UB and IB, respectively (Figure 3).

**Table 2:** The top-ten up-regulated genes in uninfected and *Opisthorchis viverrini*-infected *Bithynia siamensis goniomphalos* snails (UB and IB, respectively).

Contig ID	Size (bp)	FPKM	Top match in nr* database	ko definition**	Top match in *Biomphalaria glabrata* ***
*UB*					
CL4735.Contig4	710	19.50	Haemocyanin [*Aplypsia californica*]	Haemocyanin	Tyrosinase-domain containing protein
CL3120.Contig5	257	19.08	Uncharacterised protein	Uncharacterised	Fibrinogen-related domain containing protein
CL1900.Contig1	534	17.74	Keyhole limpet haemocyanin [*Megathura crenulata*]	Haemocyanin	Tyrosinase-domain containing protein
CL303.Contig1	683	17.54	Uncharacterised protein	Uncharacterised	Hypothetical protein
CL8571.Contig1	417	17.47	Kyelin/chordin-like protein [*Crassostrea gigas*]	Kunitz-type protease inhibitor	Kunitz-type protease inhibitor
CL1713.Contig1	1159	17.26	Hypothetical protein [*Branchiostoma floridae*]	Lectin	C-type lectin domain containing protein
CL6142.Contig2	358	17.21	Uncharacterised protein	Uncharacterised	Hypothetical protein
CL10409.Contig1	328	17.17	Uncharacterised protein	Uncharacterised	Hypothetical protein
CL800.Contig6	1116	17.13	Hypothetical protein [*Branchiostoma floridae*]	Fibropellin	Chitin-binding, multiple EGF and TSP domain-containing protein
CL8747.Contig1	1315	17.10	Hypothetical protein [*Capitella teleta*]	Fructose-biphosphatase aldolase	Fructose-biphosphate aldolase
*IB*					
CL10219.Contig4	583	17.55	Histone [*Harpegnathos saltator*]	Histone	Histone
CL636.Contig4	287	16.44	Uncharacterised protein	Uncharacterised	Hypothetical protein
CL8536.Contig2	282	16.41	Uncharacterised protein	Uncharacterised	Hypothetical protein
CL1651.Contig1	388	16.21	Wiskott-Aldrich syndrome protein [*Myotis davidii*]	Splicing factor	Thioester-containing protein
CL4675.Contig3	930	15.97	ADP-ribosylation factor [*Wuchereria bancrofti*]	ADP-ribosylation factor	ADP-ribosylation factor
CL4639.Contig1	474	16.10	Diaphanous-like protein [*Pteropus alecto*]	Formin	Relish protein
CL8803.Contig4	1103	16.07	Hypothetical protein [*Branchiostoma floridae*]	Gamma-aminobutyric acid receptor-associated protein	Hypothetical protein
CL9377.Contig2	726	16.06	Uncharacterised protein	Uncharacterised	Calcium-binding protein
CL1031.Contig2	753	15.98	Uncharacterised protein	Uncharacterised	p53-like transcription factor
CL43.Contig1	345	15.76	Dynein light-chain [*Crassostrea gigas*]	Dynein	Dynein

*non-redundant database.

**KEGG-Orthology based annotation definition.

***Based on comparisons with the preliminary *Biomphalaria glabrata* genome assembly v4.3 (http://biology.unm.edu/biomphalaria-genome/).

## Discussion

Despite the high prevalence of infection with *O. viverrini* in humans in the Greater Mekong sub-region (i.e. >90% in lowland districts of Lao PDR), and the large number of cyprinid fishes which may act as potential second intermediate hosts for this parasite [35]–[37], snail infection rates are extremely low, i.e. typically <1% [37]–[41] with peaks of ∼3% recorded in wetlands of Thailand and Lao PDR [42]. These observations lead to the hypothesis that infections of *Bithynia* snails by *O. viverrini* may stimulate a cascade of immune-molecular events similar to those induced in *Biomphalaria* spp. in response to the invasion by schistosomes, which are aimed at eliminating and/or preventing the dissemination of the infection in the snail intermediate hosts [14], [15], [43]–[45]. While knowledge of the complex mechanisms underlying the activation of immune defense pathways in *Biomphalaria* snails is expanding [46], [47], studies of the immune-molecular interactions in the *Bithynia-Opisthorchis* system are lacking. By undertaking the first large-scale comparative analysis of gene expression in UB and IB, we hoped to provide the community with a ready-to-use molecular infrastructure for in-depth studies of biological pathways specifically involved in snail-parasite interactions. An obvious limitation of this study was represented by the fact that the snail specimens subjected to RNA-Seq were collected from natural freshwater bodies, and it was therefore impossible to establish whether the uninfected *Bithynia* snails had been previously exposed to infection by *O. viverrini* and/or other parasites, or whether a previous infection had successfully been cleared. However, unlike the monoecious *Biomphalaria* spp., the dioecious nature of *Bithynia* poses some challenges for the rearing and maintenance of this snail species under experimental conditions. Indeed, it has been reported that *Bithynia* spp. specimens reared in the laboratory are smaller and morphologically altered when compared to the wild counterparts [40], [48]; since changes in body morphology are likely to correspond to alterations in gene regulation and expression, both in the steady-state and following infection by *O. viverrini*, we preferred to investigate transcriptional differences between UB and IB collected in the field.

Despite displaying significant sequence similarities to ESTs from *B. glabrata*, a significant proportion of *B. siamensis goniomphalos* DEGs (65%) could not be functionally annotated based on homology to known protein sequence data available in public databases. This finding is likely to be related to the limited number of annotated proteins available from mollusks [49], which impairs meaningful functional analysis of large-scale transcriptomic datasets. For DEGs matching available protein sequences, GO and KEGG enrichment analyses identified a range of biological pathways linked to immune responses, e.g. T and B cell signaling pathways (cf. Figure 2b), consistent with the results of previous investigations of levels of gene transcription in *Biomphalaria* snails exposed to infection by schistosomes [14], [43]–[45], [50], [51]. Amongst the immunity-associated DEGs, two proteases, a metalloprotease and a cysteine protease (cathepsin L) displayed the largest degree of up-regulation in UB when compared with IB (cf. Figure 3). The role of proteolytic enzymes, such as cathepsins, in the defense of mollusks against invading trematodes (e.g. in the breakdown of components of invading parasites) has been well documented [52], [53]. For instance, enhanced proteolytic activity has been described from extracts from *S. mansoni*-resistant compared to susceptible *B. glabrata* [53], while other studies have consistently detected transcripts encoding proteases amongst the molecules up-regulated in schistosome-resistant snail lines [14], [44]. In particular, two distinct expressed sequence tags (ESTs) encoding cathepsins L had been previously identified as highly expressed in *B. glabrata* snails susceptible to infection by the trematode parasite *Echinostoma caproni* [54] and resistant to *S. mansoni* [14], respectively. Based on the results of bioinformatic analyses of these two ESTs, Lockyer et al. [14] hypothesized that these two sequences might have represented two non-overlapping fragments of the same gene, and that the discrepancies in gene expression which had been observed followed infection by *E. caproni* and *S. mansoni* might have been due to “the different response elicited by two different parasite species” [14]. Comparative analyses between the abovementioned *Biomphalaria* ESTs and the cathepsin L-encoding sequence identified in the present study suggest that the latter transcript is indeed distinct from the two previously identified (data available from the corresponding author upon request). Moreover, in addition to the transcript encoding a ‘novel’ cathepsin L, a molecule encoding a ficolin was identified amongst the most highly expressed transcripts in UB (cf. Figure 3). Ficolins are a group of lectin-like defense proteins that, in vertebrates, are known to recognize carbohydrate molecules on pathogens and/or apoptotic and necrotic cells [55]. Structurally, ficolins consist of a small N-terminal domain, a collagen-like domain and a fibrinogen-like domain similar to the C-terminal domains of the beta and gamma chains of fibrinogen [56]. These molecules, which belong to a large protein family known as Fibrinogen-Related Proteins (FREPs), play key roles in the innate immunity of both vertebrate and invertebrate organisms [57]. In particular, in mollusks, FREPs are believed to exert ‘antibody-like’ properties in biological mechanisms of recognition and binding of invading pathogens [58], [59]. In *B. glabrata*, transcription of FREP-encoding genes is enhanced after exposure of the snail to both *S. mansoni* and *E. paraensei* [43], [60], [61]; furthermore, FREPs have been shown to bind sporocysts of both *S. mansoni* and *E. paraensei*, thus actively participating in molecular events linked to defense against pathogens [62]. A mucin and an immunoglobulin receptor homologue were also amongst highly transcribed molecules in UB (cf. Figure 3). While the up-regulation of transcripts encoding mucins could be attributed to a role of these proteins in the establishment of a physical defense barrier against invading pathogens [63], the detection of an immune receptor-like molecule amongst the DEGs is interesting, since mollusks lack adaptive immunity. However, in a previous study, Bayne and co-workers [64] demonstrated that hemocytes of *B. glabrata* were unable to recognize IgG-bound antigens of *S. mansoni* sporocysts, thus providing evidence for the existence of specific antigen-binding sites on snail hemocytes [64]. Results from the present study support this hypothesis.

While it is tempting to speculate that the identification of homologues of a protease, a FREP and an immunoglobulin-receptor amongst the most highly expressed transcripts in UB (collected from the same natural water bodies as IB) may be associated with a ‘successful’ defense response against *O. viverrini*, which ultimately resulted in prevention or clearance of the infection (similarly to resistant strains of *B. glabrata*), such a hypothesis requires rigorous testing.

Amongst the DEGs that could be linked to pathways associated with immune responses, actins and heat-shock proteins (HSPs) were identified amongst the molecules with the largest degree of up-regulation in IB (cf. Figure 3). The function/s of HSPs in stress responses of hemocytes of *B. glabrata* in response to schistosome infection has been documented [65], [66]; conversely; the role/s played by actins in snail-parasite relationships is unclear. Since defense against pathogens in mollusks mainly relies on the migration of circulating hemocytes to the sites of infection and tissue damage, it could be hypothesized that the relative increase in transcription of actin-encoding genes might reflect a general increase in cellular motility, as well as phagocytosis and cell trafficking. Besides actins and heat-shock proteins, the absence of key immune-regulatory molecules amongst the most-significantly up-regulated genes in IB remains to be investigated; one possible explanation could be that, similarly to schistosome parasites infecting susceptible *Biomphalaria* spp. [14], *O. viverrini* manipulates the innate defense system of susceptible *Bithynia*, effectively resulting into the inability of the hemocytes to recognize the parasite and/or into the suppression of the snail humoral responses against the parasite invasion. This hypothesis is also supported by the fact that, out of the DEGs identified in this study, the vast majority (i.e. ∼90%) were molecules whose levels of transcription were up-regulated in UB, thus reinforcing a potential role of *O. viverrini* in mechanisms of immune suppression in susceptible snails [14].

Taken together, the similarities in gene expression profiling between parasite-free specimens of wild-caught *B. siamensis goniomphalos* examined in the present study and schistosome-resistant populations of *B. glabrata*, and between infected *Bithynia* and schistosome-susceptible *Biomphalaria*, lead to the speculation that an *O. viverrini*-resistant population of *Bithynia* snails may occur in natural freshwater bodies of Northeast Thailand. However, it must be pointed out that the snail specimens herein examined were screened for the presence of *O. viverrini* DNA only; therefore, the occurrence of underlying infections by other snail pathogens in both UB and IB cannot be excluded. Ultimately, in order to test the hypothesis that *O. viverrini*-resistant *B. siamensis goniomphalos* may occur in Thailand, the development of adequate and efficient methods to successfully rear and maintain populations of this snail species under laboratory conditions is necessary. Once an optimal rearing protocol is established, future studies could investigate changes in gene expression of key genes at different time points following experimental infection of *Bithynia* specimens with *O. viverrini*; such studies will allow to further explore the role/s of selected molecules, such as proteases and actins, in the cascades of molecular events that determine susceptibility or resistance of snails to trematode parasites.

A large collaborative project aimed at generating the first reference whole genome sequence of *B. glabrata* is currently underway (http://biology.unm.edu/biomphalaria-genome/). Once completed, the *B. glabrata* genome will represent an unprecedented resource for in-depth studies of snail-trematode interactions, as well as for comparative analyses between and among species acting as intermediate hosts of parasitic flukes, and for the discovery of the molecular and evolutionary mechanisms associated to the selection of parasite-resistant snail populations [67], [68]. Similarly, the wealth of molecular data that is currently available for *O. viverrini* [10], [11], [69], together with future efforts to define its entire genome sequence, will set the basis for the identification of genes and gene products essential for the recognition and infection of susceptible snail hosts. Taken together, these advances will provide the scientific community with new tools for the development of strategies to control the spread of opisthorchiasis and *Opisthorchis* induced-CCA.

## Supporting Information

Table S1 The annotated transcriptome of *Bithynia siamensis goniomphalos* and corresponding sequence data. Summary of annotation data linked to individual assembled transcripts. Abbreviations: non-redundant protein database (Nr), non-redundant nucleotide database (Nt), SwissProt database (SwissProt), Clusters of Orthologous Groups of proteins (COG), Kyoto Encyclopaedia of Genes and Genomes (KEGG); Gene Ontology (GO).
(ZIP)

Table S2 Differentially expressed genes (DEGs) inferred from the transcriptomes of uninfected and *Opisthorchis viverrini*-infected *Bithynia siamensis goniomphalos* snails (UB and IB, respectively). Abbreviations: fragments per kilobase of exon per million fragments (FPKM), false discovery rate (FDR), non-redundant protein database (Nr), non-redundant nucleotide database (Nt), SwissProt database (SwissProt), Clusters of Orthologous Groups of proteins (COG), Kyoto Encyclopaedia of Genes and Genomes (KEGG); Gene Ontology (GO).
(XLS)
